# Machine Learning–Based Biomechanical and Clinical Assessment of the Five Times Chair Rise Test in Older Adults: Sensor-Based Functional Aging Study

**DOI:** 10.2196/94597

**Published:** 2026-11-09

**Authors:** Anna Schumacher, Ole Meyer, Vincent Quinten, Andreas Hein, Rebecca Diekmann

**Affiliations:** 1 Assistance Systems and Medical Device Technology, School VI - Medicine and Health Sciences, Carl von Ossietzky Universität Oldenburg, Ammerländer Heerstraße 140, Oldenburg, Lower Saxony, 26129, Germany, 49 4417984630; 2 OFFIS - Institute for Information Technology, Oldenburg, Lower Saxony, Germany; 3 Junior Research Group “Nutrition and Physical Function in Older Adults”, School VI - Medicine and Health Sciences, Carl von Ossietzky Universität Oldenburg, Oldenburg, Lower Saxony, Germany

**Keywords:** healthy aging, digital health, machine learning, clustering, biomechanics, functional assessment, mobility, chair rise, older adults, frailty, movement analysis

## Abstract

**Background:**

Maintaining functional ability is essential for healthy aging. The Five Times Chair Rise Test (5CRT) is widely used to assess lower-extremity function, yet conventional stopwatch timing provides limited insight into movement quality and compensatory strategies. Sensor-based biomechanics combined with machine learning (ML) enables detailed analysis of movement dynamics and supports digital health approaches for early detection and monitoring of functional decline. However, the clinical relevance of ML-based 5CRT movement patterns and their relationship to established functional outcomes and aging trajectories remains insufficiently understood.

**Objective:**

This study aimed (1) to classify 5CRT movement patterns using an established dynamic time warping (DTW)-based clustering workflow; (2) to examine associations between ML-based movement clusters and multidimensional indicators of strength, mobility, and functional performance across 24 months; and (3) to evaluate whether ML-based movement classification provides clinically meaningful differentiation beyond conventional stopwatch-based 5CRT timing within a digital health assessment context.

**Methods:**

In this longitudinal observational study, community-dwelling adults aged ≥70 years completed standardized functional assessments at baseline, 6 months, and 24 months, including Handgrip Strength, Stair Climb Power Test, Timed “Up & Go” Test, Short Physical Performance Battery (including 5CRT), 4-meter Gait Speed, Balance Testing, 6-minute Walking Test, and Countermovement Jump. The assessments were recorded using a force plate and depth camera to capture biomechanical time-series data. DTW and k-means clustering were applied to ground reaction force signals to identify movement patterns.

**Results:**

Among 172 participants included in the final analysis (mean age 76.9, SD 3.40 years; 96/172, 56% female), 2 distinct movement clusters were identified. Cluster 1 demonstrated faster chair rise performance and lower stabilization forces, whereas cluster 2 exhibited slower execution and increased stabilization demands. Across baseline, 6-month, and 24-month follow-up assessments, cluster 1 consistently showed higher muscle strength, greater leg power, faster mobility, higher gait speed, and longer walking distance than cluster 2 (all *P*<.01). At 24 months, large between-cluster differences were observed for the Timed “Up & Go” Test, 5CRT duration, gait speed, 6-minute walk distance, and Short Physical Performance Battery score. Conventional stopwatch scoring categorized most participants as high functioning, whereas ML-based clustering revealed substantial functional heterogeneity and clinically meaningful information not reflected by timing alone.

**Conclusions:**

ML-based analysis of sensor-based chair rise performance identifies functional performance profiles beyond conventional timing metrics. Integrating biomechanical movement analytics into digital health assessment frameworks may improve early detection of mobility decline and support longitudinal monitoring and personalized intervention planning in older adults.

## Introduction

Striving for healthy aging and maintaining independence among older adults is a critical challenge in our rapidly aging global population [1,2]. The World Health Organization defines healthy aging as the process of developing and maintaining functional ability that enables well-being in older age [2,3]. However, functional ability tends to decline with age [4], impacting mobility, autonomy, and quality of life.

Functional ability is characterized by an individual’s capacity to perform tasks essential for independent living, such as meeting basic needs, making decisions autonomously, maintaining social relationships, and moving independently [3,5-7]. Identifying the threshold at which age-related decline compromises mobility and independence, and understanding compensatory movement strategies individuals adopt to overcome these deficits, is a key focus of healthy aging research [4]. Early detection and quantification of these compensations can inform timely interventions, helping to prolong independence and reduce care needs.

The Five Times Chair Rise Test (5CRT) is a widely used clinical assessment to evaluate lower-extremity functional ability and strength in older adults [8]. Conventionally, performance is measured by timing the duration of 5 sit-to-stand cycles using a stopwatch, yielding a single temporal metric [8,9]. While practical, this approach does not fully capture the complexity and nuance of the movement patterns involved, nor does it exploit the rich biomechanical data potentially available from force and motion analyses [10,11]. Older adults with physical impairments, such as muscle weakness, often develop compensatory strategies—including altered center-of-mass trajectories or increased reliance on stabilization forces—that may enable task completion but mask underlying deficits [4]. Detailed biomechanical characterization of the 5CRT phases can reveal early functional decline and compensatory adaptations not reflected in timing metrics alone.

Meyer et al [10] introduced a machine learning (ML)–based workflow that combines dynamic time warping (DTW) and k-means clustering to classify 5CRT movement patterns from multidimensional force plate data in older adults. DTW was used to compensate for nonlinear temporal variability in sit-to-stand execution processes. Signal comparisons are not based on absolute duration. Rather, DTW aligns force trajectories according to their morphological characteristics. This allows k-means clustering to identify groups with similar movement patterns despite differences in execution speed. Meyer et al [10] demonstrated that unsupervised clustering of DTW-derived features can reveal distinct movement strategies that are invisible to stopwatch timing [10]. This established the methodological feasibility of the approach. However, Meyer et al [10] focused on discovering and describing biomechanical patterns without investigating how these movement clusters relate to established clinical indicators of physical function, mobility, and frailty.

This study uses the DTW clustering framework of Meyer et al [10] to ensure methodological comparability and reproducibility. The innovation lies in its integration with an extensive battery of validated clinical and functional assessments, including the Handgrip Strength (HGS) test, the Stair Climb Power Test (SCPT), the Timed “Up and Go” (TUG) test, the Short Physical Performance Battery (SPPB), including the 5CRT, the 4-meter Gait Speed (4mGS) test, and the Balance Test (BT), as well as the 6-minute Walking Test (6minWT) and the Countermovement Jump (CMJ). By directly correlating DTW-derived 5CRT movement clusters with these multidimensional performance measures, this study addresses a critical gap.

Preliminary analyses by Meyer et al [10] identified 2 distinct DTW-derived movement clusters with clear biomechanical and functional performance characteristics in a cohort of community-dwelling older adults. On average, cluster 1 was characterized by faster 5CRT completion times, lower vertical ground reaction forces (GRFs) during the stabilization phase, and higher mobility and strength assessment scores, indicating greater physical function and more efficient postural control. Conversely, cluster 2 exhibited slower chair rise times, higher stabilization forces, and poorer performance in mobility and functional tests. This suggests diminished lower-extremity power, potential balance limitations, and an increased risk of mobility-related decline. Contextualizing these biomechanically defined performance profiles within established geriatric assessments allows for a clinically meaningful classification, extending the applicability of ML-based motion analysis to real-world screening and intervention planning.

This work is not a methodological comparison but rather a clinical validation and transfer study. By applying the DTW and k-means clustering workflow by Meyer et al [10], methodological comparability is ensured, enabling validation of their findings in a longitudinal cohort while extending the analysis to its clinical implications across multiple functional domains.

The specific aims of this study are:

1. To classify 5CRT movement patterns using the established DTW and k-means workflow by Meyer et al [10], ensuring methodological comparability and enabling the investigation of aging trajectories in sensor-based movement characteristics.
2. To examine the association between DTW-defined 5CRT movement clusters and key indicators of lower extremity strength, mobility, exercise capacity, and functional performance as measured by HGS, SCPT, TUG, SPPB, 5CRT, 4mGS, BT, 6minWT, and CMJ, thereby exploring their relationship to interindividual variability and aging trajectories.
3. To evaluate whether ML-based 5CRT movement pattern classification provides clinically meaningful differentiation of functional status beyond conventional stopwatch-based 5CRT timing, and whether this approach contributes to capturing and monitoring functional aging trajectories over time.

This combined approach of traditional stopwatch timing and sensor-based, ML-driven movement pattern classification is used to create actionable functional performance profiles. These profiles could aid in the early identification of mobility decline, inform intervention planning, and ultimately support healthy aging trajectories. Additionally, linking the analysis to established clinical outcomes connects biomechanical analytics to the decision-making requirements of geriatric care and digital health applications.

## Methods

### Study Participants

The study included a cohort of healthy, community-dwelling older adults [12,13]. Participants were recruited across diverse settings, including sports clubs, senior centers, music societies, rehabilitation sports facilities, and physiotherapy departments, as well as through newspaper advertisements. Functional geriatric assessments were systematically conducted under strict inclusion criteria. Participants had to be at least 70 years old and live in the community (Figure 1). They could not be included in the study if they had major acute morbidities such as pulmonary, renal, or cardiac diseases. They had to be able to climb 10 flights of stairs without difficulty and be self-sufficient enough to attend assessment sessions. They could not have pacemakers or other electronic implants. They had to be able to complete a TUG test in less than 20 seconds.

Prior to the assessment, participants underwent a telephone interview and received the written informed consent form at least one week in advance. Written informed consent was obtained from all participants prior to their participation. Additionally, a thorough medical history was collected, including a semistructured questionnaire on health status (eg, hypertension, history of stroke, chronic conditions such as diabetes mellitus and chronic obstructive pulmonary disease (COPD), falls, and general health) and a review of current medications. For safety reasons, blood pressure was measured, and participants with a reading exceeding 180/95 mm Hg were excluded from the study.

**Figure 1.**
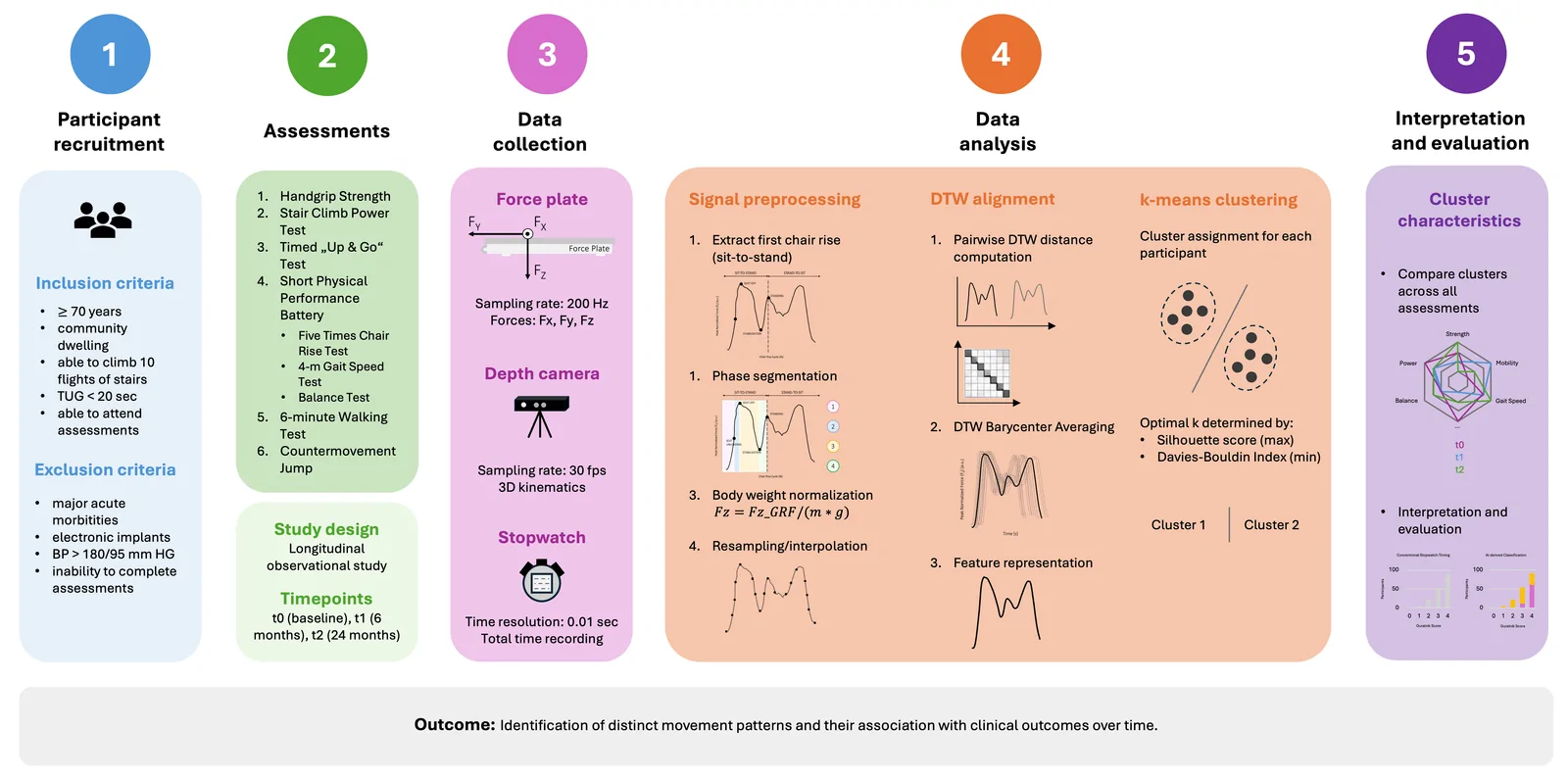
Graphical workflow of the study, summarizing participant recruitment, clinical assessment, biomechanical data acquisition, signal processing, dynamic time warping alignment, and machine learning–based clustering analysis. BP: blood pressure; DTW: dynamic time warping; TUG: Timed “Up & Go.”

### Study Design

This longitudinal observational study included 2 follow-up assessments (Figure 1). Eligible participants were evaluated at the Carl von Ossietzky Universität Oldenburg study center in Germany. Three assessment time points were conducted: (1) baseline (t0), (2) 6-month (t1) follow-up, and (3) 24-month (t2) follow-up.

Prior to each assessment, participants’ height and weight were measured using a stadiometer. Participants then completed a standardized battery of the following physical performance tests (Table 1), administered consistently according to standardized protocols.

**Table 1.** Overview of physical performance tests applied in the study and corresponding test procedures.

Physical performance test	Test procedure
1. HGS^a^	
Reduced HGS = frailty criteria	Measured with a JAMAR handheld dynamometer Participant seated during the test Both hands tested alternately, 3 trials per hand Highest maximum value from 3 trials (left or right hand, whichever is stronger) is used Strength values evaluated based on age and BMI according to [14]
2. SCPT^b^	
Leg power	Participant climbs a flight of 10 stairs as quickly as possible without running or skipping steps Time to climb the stairs is recorded Body mass and total stair height are measured Related power was calculated according to [15]: *P*=m x g x h/t (W), m=body mass (kg), g=9,81 m/s^2,^ h=height (m), *t*=time (s)
3. TUG^c^	
Functional mobility	Participant sits with back against the chair The signal “Go” starts the test Participant stands up, walks 3 meters to a marked line, turns around, walks back, and sits down again Time is measured from standing up to sitting down Assistive devices allowed, but no physical help from others
4. SPPB^d^	
4.1. lower extremity physical function	The test battery included the 5CRT^e^, 4mGS,^f^ and BT^g^ The time (in seconds) it takes to complete each test, as well as the cumulative score, are used to evaluate the results according to [8]
** **4.2. 5CRT	Participant sits on a chair, and a signal starts the test Participant stands up and sits down 5 times in a row as quickly as possible Total time for all 5 repetitions is recorded with a stopwatch
4.3. 4mGS	Participant walks a marked 4 meter distance at a normal pace Time needed is recorded with a stopwatch
** **4.4. BT	Semi-tandem and tandem stand Participant holds specific positions (semi-tandem or full tandem stand) for a set of time Ability to maintain position without support is assessed
5. 6minWT^h^	
Functional exercise capacity Endurance performance	Participant walks up and down a 20 meter corridor for 6 minutes at the normal pace Total distance covered is recorded in meters Tester accompanies participant for safety reasons Time needed is measured with a stopwatch
6. CMJ^i^	
Functional performance	Participant stands with feet at hip width and knees bent at about 90° at starting position on a ground reaction force platform (AMTI AccuPower, 200 Hz) When jumping as high as possible, the knees are straightened (no leg tuck or arm instruction) One preliminary jump: 3 test jumps with one-minute rest; weight is measured before first jump Maximum jump power (W) was calculated from the 3 best jumps The CMJ test was conducted at t0 and t1, but not at t2 for safety reasons

a HGS: Handgrip Strength.

b SCPT: Stair Climb Power Test.

c TUG: Timed “Up & Go” Test.

d SPPB: Short Physical Performance Battery.

e 5CRT: Five Times Chair Rise Test.

f 4mGS: 4-meter Gait Speed test.

g BT: Balance Test.

h 6minWT: 6-minute Walking Test.

i CMJ: Countermovement Jump.

### Assessments of Physical Function

To ensure methodological comparability with Meyer et al [10], the DTW and k-means clustering workflow was applied to the 5CRT at the 24-month follow-up (t2) assessment. Recent methodological advances have demonstrated the potential of using the biomechanical data collected during the 5CRT for time series analysis and unsupervised clustering. This allows for the identification of clinically relevant functional subgroups [10]. Thus, the 5CRT offers a chance to integrate quantitative biomechanical parameters with established clinical assessment frameworks. The detailed protocol for the 5CRT used in this study is described below.

Participants performed the 5CRT by rising from a chair to a fully stable standing position and sitting down again as quickly as possible, following the protocol described by Guralnik et al [8], with them being seated upright in the center of a height-adjustable chair without armrests. Participants started with their knees flexed at a 90-degree angle (verified by a goniometer) and their feet placed shoulder-width apart on the force plate positioned directly in front of the chair. Throughout the test, arms were crossed in front of the chest to prevent the use of arm support. A verbal command initiated the test. Participants were instructed to stand up completely (sit-to-stand) and then sit back down with their buttocks touching the chair (stand-to-sit). They repeated this movement cycle 5 times consecutively without using their arms. The total time to complete the 5 rises was recorded using a Schütt PC-90/60 Memory stopwatch with a time resolution of 1/100 second and a memory function for intermediate times (up to 60 splits). It was then scored according to Guralnik et al [8] (Table 2). Timing started with the command and stopped once the participant completed the fifth rise and made full contact with the back of the chair. All measurements were conducted by trained physiotherapists to ensure consistency.

**Table 2.** The 5CRT^a^ is scored based on the measured test duration, as used in the SPPB^b^ as a frailty measure according to Guralnik et al [8]. A higher score indicates better performance in the 5CRT.

5CRT^a^ durations (in seconds)	Scores
≤11.19	4 points
11.20‐13.69	3 points
13.70‐16.69	2 points
16.70‐60	1 point
>60 or unable to complete the 5CRT	0 points

a 5CRT: Five Times Chair Rise Test.

b SPPB: Short Physical Performance Battery.

### Data Collection

During the test, external GRFs were recorded at a sampling rate of 200 Hz using a calibrated AMTI AccuPower force plate (Figure 1). Participants stood on this plate with both feet. The plate was not moved during or between the assessments. Forces were captured in 3 directions during the assessment: medial-lateral (Fx), anterior-posterior (Fy), and vertical (Fz). Before each assessment, the force plate was zeroed and calibrated by inputting participant-specific data such as sex, height, and weight. Additionally, a Microsoft Kinect V2 depth camera (30 fps) recorded movements in 3 dimensions, enabling correlation of kinematic data with kinetic force measurements for comprehensive biomechanical signal interpretation.

### Data Analysis

Data analysis was performed using Python (Python Software Foundation) [16], including the *tslearn* package [17]. Raw force plate and motion capture data were synchronized, visually inspected for artifacts, and segmented into individual sit-to-stand trials [10] (Figure 1). Using the standardized, clinically established 5CRT as the basis for the clustering process ensures compatibility with widely used frailty and functional assessments and allows the use of detailed kinetic and kinematic data available from force plate and motion capture systems. This strategy allows to characterize the heterogeneity of functional ability in older adults, supporting a more individualized, data-driven approach to understanding and monitoring the decline of age-related mobility.

The sit-to-stand movement was analyzed by segmenting it into 4 distinct phases based on the model by Schenkman et al [18] (Figure 2):

1. Phase 1: The “Flexion Momentum Phase” begins with movement initiation and continues until just before the participant’s buttocks lift off the chair (seat-off). This phase is characterized by trunk and pelvic flexion, as well as anticipatory postural adjustments primarily involving the head, arms, and trunk.
2. Phase 2: The “Momentum Transfer Phase,” spans from seat-off to maximum ankle dorsiflexion. During this phase, the body’s center of mass shifts significantly forward and upward.
3. Phase 3: The “Extension Phase” starts immediately after maximum ankle dorsiflexion and concludes with full hip and knee extension.
4. Phase 4: The “Stabilization Phase” marks the final upright standing position, with the participant fully balanced.

To address the temporal variability and nonlinear time distortions inherent in human movement sequences, DTW was applied as a time series similarity measure [10]. For each participant, the vertical GRF signal (Fz) was peak-normalized to body weight, resampled to a common temporal length, and used as the input time series for DTW-based comparison. DTW aligns temporal force profiles to reveal latent spatiotemporal patterns within participant-specific movement cycles. This enables comparisons independent of the absolute duration of the chair rise.

The clustering model was based on previously derived centroids by Meyer et al [10] using k-means clustering with DTW Barycenter Averaging (DBA) as implemented in *tslearn* [17]. Clusters were assigned based on each participant’s first chair rise performance. Therefore, for initial cluster assignment, only the first chair rise from the 5CRT sequence was used. This was done to minimize potential influences of fatigue, learning effects, or adaptation on movement strategy that could occur over successive repetitions. Subsequent chair rises were classified using the same model to evaluate stability and changes in movement patterns across repetitions. To reduce the risk of local minima, the clustering procedure was repeated 10 times with different centroid initializations, and the maximum number of iterations was set to 100 [10].

**Figure 2.**
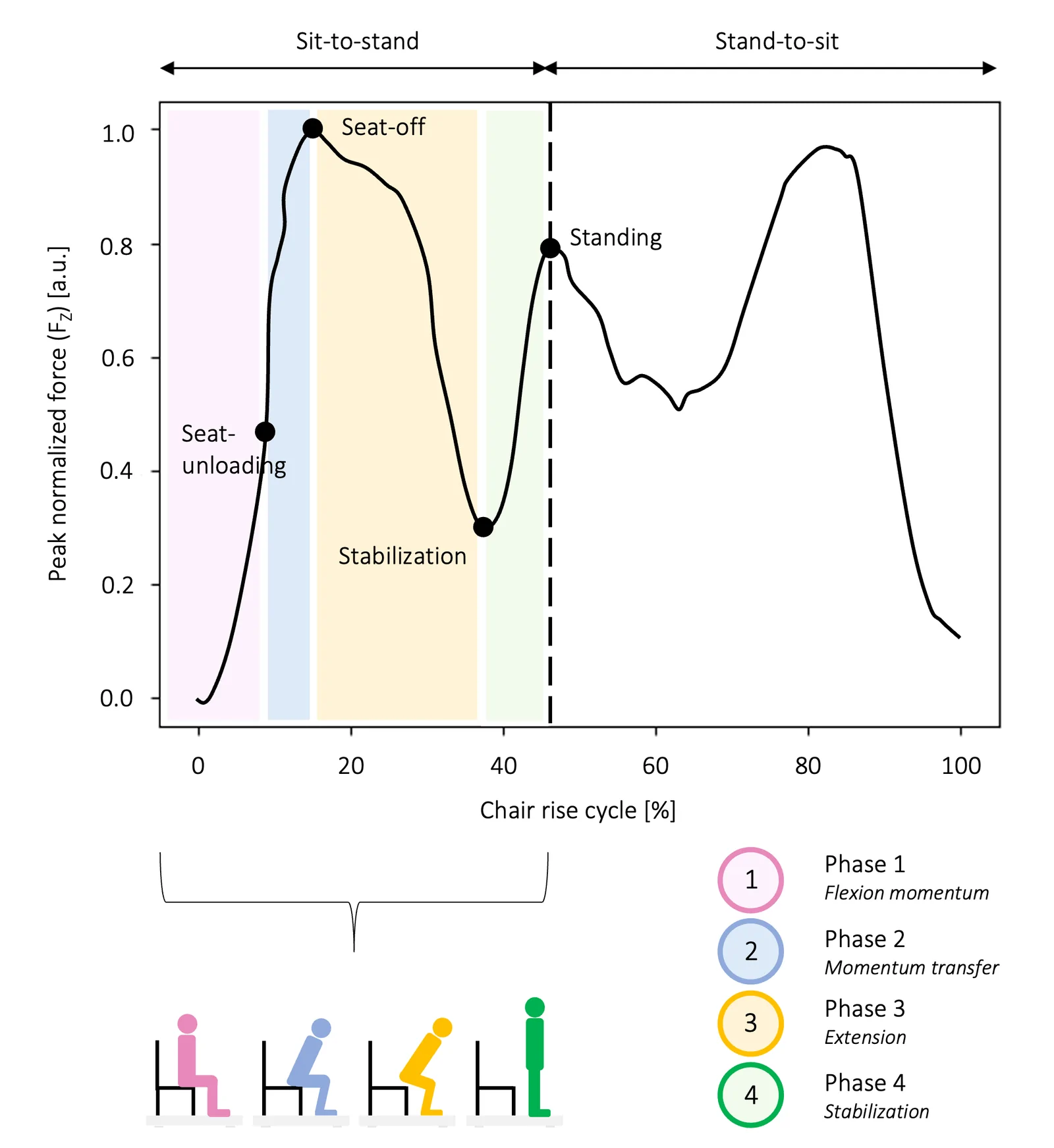
The different phases of the sit-to-stand part of the Five Times Chair Rise Test, as defined by Schenkman et al [18], can be seen in the Fz component of the ground reaction force data of a random participant. Phase 1 (flexion momentum): initiation of the movement. Phase 2 (momentum transfer): seat unloading until maximum dorsiflexion is reached. Phase 3 (extension): the buttocks leave the chair and the whole body moves upwards. Phase 4 (stabilization): The hip and knee joints are fully extended. The sit-to-stand transition is completed when the participant stands fully upright. The color-coded schematics illustrate the corresponding body posture during each phase of the sit-to-stand cycle according to Schenkman et al [18].

### Evaluation

The evaluation of unsupervised clustering, which is inherently challenging due to its lack of predefined labels, required the application of multiple validation metrics (Figure 1). Silhouette analysis was initially used to evaluate the separation of clusters by measuring the mean intra versus intercluster distances for k=2‐10 (*tslearn* implementation following Rousseeuw [17]). Higher values in this case indicate better separation, with a score near to 1 denoting well-defined clusters, and a score near to 0 indicating partial overlap, and a negative score indicates misclassification. The k-value that yielded the highest silhouette score was selected for clustering.

Subsequent assessment of cluster validity was conducted using the Davies-Bouldin Index [19], a quantitative metric that calculates the ratio of intracluster compactness to intercluster separation.

The Davies-Bouldin Index [19] is defined as follows:


$$\bar{R}\equiv \frac{1}{N}\sum_{i=1}^{k}\underset{j\neq i}{\max}\left(\frac{S_{i}+S_{j}}{M_{ij}}\right)$$

where *k* is the number of clusters, *S_i_* and *S_j_* denote the within-cluster scatter of clusters *i* and j, and *M_ij_* represents the distance between the centroids of clusters *i* and j. Lower values indicate greater homogeneity and separation between clusters.

### Statistical Analysis

The Shapiro-Wilk test was used to test the normal distribution of the force and duration values obtained from the chair rise dataset. Since the dataset contained nonnormally distributed data, the nonparametric Mann-Whitney *U* test was used to compare the clustered 5CRT groups. Statistical significance was defined as *P*<.01. The analysis was performed in Python [16] using the *SciPy* package.

Then, the 5CRT clusters were correlated with the conducted physical performance tests (Table 1) across the 3 time points (baseline: t0, 6 months: t1, and 24 months: t2). Independent samples *t* tests (2-tailed) were used to compare continuous outcomes between the 2 clusters at t0, t1, and t2. For categorical variables, the Pearson chi-square test was applied. Where normality assumptions were not met, the Mann-Whitney *U* test served as a nonparametric alternative. Normality and homogeneity of variance were assessed using the Shapiro-Wilk test and the Levene test, respectively. Statistical significance was set at *P*<.05, *P*<.01, and *P*<.001. Effect sizes were reported as Cohen *d* with 95% CIs. To compare effect sizes across assessments and time points, Cohen *d* values were visualized in a radar plot. All analyses were performed using IBM SPSS Statistics for Windows, version 30.0.0 (171).

### Ethical Considerations

The study was approved by the Medical Ethics Committee of Hanover Medical School in Germany (approval number 6948) and was conducted in accordance with the Declaration of Helsinki [20], and all relevant guidelines and regulations. All participants provided written informed consent. Participant data were pseudonymized, and identifying information was stored separately from the study data. Access to the data was restricted to authorized study personnel, and electronic data were stored on password-protected or access-restricted systems. During transcription, identifying information was removed or modified. The participants identification list was deleted after the study was completed. Participants received reimbursement for travel expenses but no other compensation.

## Results

### Characteristics of Study Participants

A total of 251 participants were initially enrolled at baseline (t0). Four participants dropped out by the 6-month follow-up (t1), and an additional 19 participants were lost to follow-up by 24 months (t2; Table 3).

The following sections present the results in the order of the predefined study aims.

**Table 3.** Characteristics of study participants at baseline (t0), 6-month (t1), and 24-month (t2) follow-up assessments^a^.

Assessments	Study participants (N)	Age (years)		Sex	
		Range	Mean (SD)	Female n/N (%)	Male n/N (%)
Baseline (t0)	251	70-87	75.10 (3.89)	148/251 (59)	103/251 (41)
6 months (t1)	247	70-88	75.87 (3.84)	146/247 (59)	101/247 (41)
24 months (t2)	228	71-89	77.23 (3.78)	132/228 (58)	96/228 (42)

a Values are presented as mean (SD), including minimum (Min), and maximum (Max) values for age (years) and sex (female and male).

### Classification of 5CRT Movement Patterns Using ML-Based Clustering

At t2, 228 participants were included in the assessment, and 191 of these participants completed the 5CRT. Nine participants were then excluded from data analysis due to errors in the recorded measurement data, and 10 participants were excluded because they failed to complete the required 5 chair rises. Consequently, the final analytic 5CRT sample at t2 comprised 172 participants with a mean age of 76.90 (SD 3.40) years (Table 4). Among them, 96 (56%) were female and 76 (44%) were male.

A total of 860 chair rises from the t2 assessment were categorized into 2 clusters (clusters 1 and 2) using k-means clustering based on the DBA algorithm [10]. Silhouette analysis indicated that 2 clusters were the optimal solution (Davies-Bouldin index~0.92). The 2 resulting clusters were defined by the applied force (Fz) during the chair rise process. Figure 3 shows the soft-DTW barycenter to visualize the different clusters.

**Table 4.** Study participants’ characteristics at 24-month follow-up (t2) measured via stopwatch.

Characteristics	Mean (SD)	Range	
Age (years)	76.90 (3.40)	71-89	
Weight (kg)	75.10 (13.40)	48.10-126	
Height (m)	1.67 (0.09)	1.45-1.91	
5CRT^a^ stopwatch duration (seconds)	11.44 (2.49)	6.44-20.21	

a 5CRT: Five Times Chair Rise Test.

**Figure 3.**
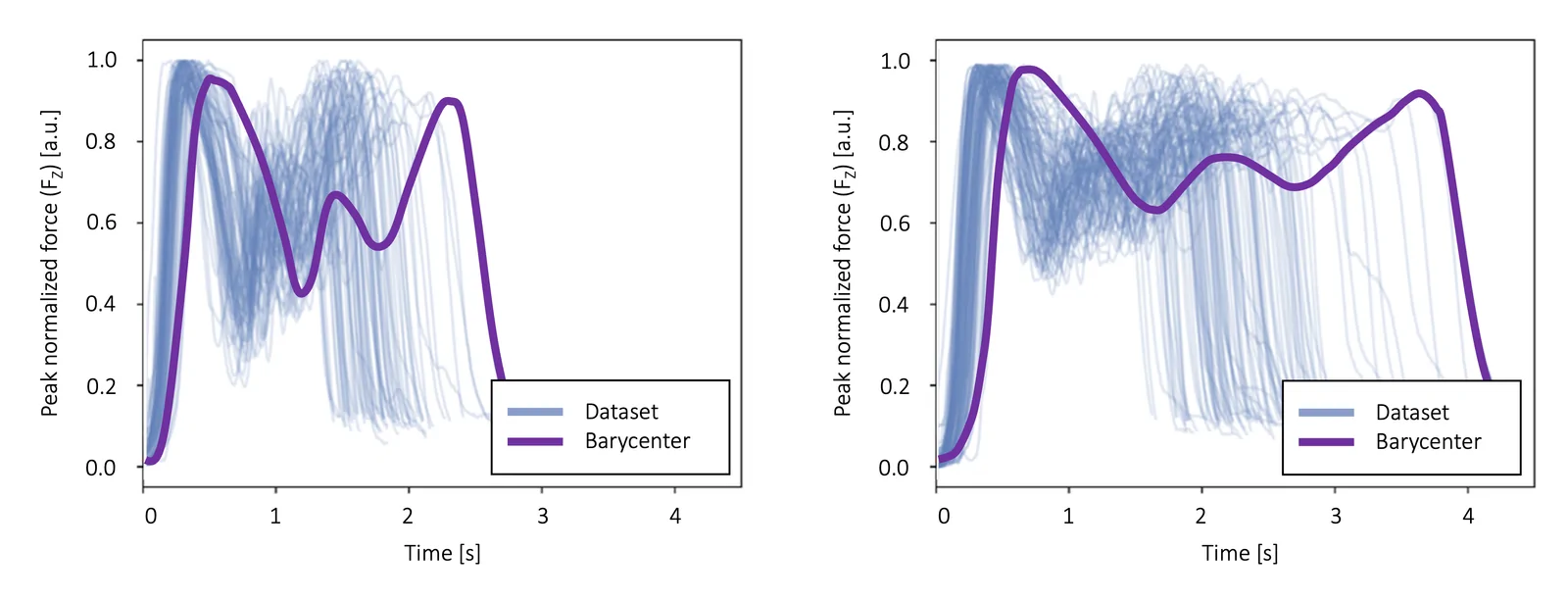
Dataset and barycenter of cluster 1 (left) and cluster 2 (right) after k-means clustering according to Meyer et al [10].

K-means clustering assigned 72 (42%) participants to cluster 1 and 100 (58%) participants to cluster 2 (Tables 5–7). Men and women were distributed unevenly across clusters: 47% (n=40) of male participants were assigned to cluster 1, while 53% (n=44) were categorized in cluster 2. Overall, 37% (n=32) of the female participants were assigned to cluster 1, while 63% (n=56) were assigned to cluster 2.

**Table 5.** Demographic and physical performance outcomes for participants in DTW^a^-defined 5CRT^b^ movement clusters (cluster 1 and 2) according to Meyer et al [10].

Variables at t0 (baseline)	Cluster 1 (n=72)	Cluster 2 (n=100)	*P*^c^ value	Effect size, Cohen *d* (95% CI)
Age (years), mean (SD)	74.40 (2.70)	75.30 (3.90)	.10	–0.259 (–0.563 to 0.045)
Sex, n (%)			.19^d^	
Male	36 (50)	40 (40)		
Female	36 (50)	60 (60)		
BMI (kg/m^2^), mean (SD)	25.64 (2.90)	27.97 (4.09)	<.001	–0.640 (–0.949 to –0.328)
HGS^e^ (kg), mean (SD)	32.41 (9.88)	28.07 (9.60)	.004	0.447 (0.140 to 0.753)
SCPT^f^ (W), mean (SD)	236.15 (51.30)	214.66 (47)	.005	0.440 (0.133 to 0.746)
TUG^g^ (s), mean (SD)	7.42 (0.95)	8.74 (1.44)	<.001	–1.047 (–1.368 to –0.723)
SPPB^h^ total score, mean (SD)	11.35 (0.75)	10.93 (1.10)	.006	0.429 (0.122 to 0.735)
5CRT stopwatch duration (s), mean (SD)	11.15 (2.07)	12.80 (3.30)	<.001	–0.581 (–0.889 to –0.271)
4mGS^i^ (s), mean (SD)	2.44 (0.30)	2.87±0.42)	<.001	–1.167 (–1.493 to –0,839)
‍4mGS (m/s), mean (SD)	1.66 (0.22)	1.42 (0.20)	<.001	1.190 (0.860 to 1.517)
6minWT^j^ distance (m), mean (SD)	490 (60.27)	421.21 (60.34)	<.001	1.141 (0.813 to 1.465)
‍Semitandem stand (points), mean (SD)	1 (0)	0.99 (0.10)	.40^k^	
‍Tandem stand (points), mean (SD)	1.99 (0.12)	1.92 (0.32)	.06^l^	
CMJ^m^ height (cm), mean (SD)	13.07 (4.71)^n^	9.55 (4.23)^o^	<.001	0.793 (0.463 to 1.121)
CMJ power (W), mean (SD)	2050.17 (577.12)^n^	1816.86^o^ (482.32)	.007	0.445 (0.124 to 0.765)

a DTW: dynamic time warping.

b 5CRT: Five Times Chair Rise Test.

c *P* values are derived from independent samples *t* tests (highly significant: *P*<.001, significant: *P*<.01, marginally significant: *P*<.05).

d Derived from Pearson chi-squared test for categorical variables: *χ*2_1_=1.697

e HGS: Handgrip Strength.

f SCPT: Stair Climb Power Test.

g TUG: Timed “Up & Go.”

h SPPB: Short Physical Performance Battery.

i 4mGS: 4-meter Gait Speed test.

j 6minWT: 6-minute Walking Test.

k Derived from Mann-Whitney *U* test for nonparametric data: *U*=3564.

l Derived from Mann-Whitney *U* test for nonparametric data: *U*=3361.5.

m CMJ: Countermovement Jump.

n n=66 for cluster 1.

o n=91 for cluster 2.

**Table 6.** Demographic and physical performance outcomes for participants in DTW^a^-defined 5CRT^b^ movement clusters (cluster 1 and 2) according to Meyer et al [10].

Variables at t1 (6 months)	Cluster 1 (n=72)	Cluster 2 (n=100)	*P^c^* value	Effect size, Cohen *d* (95% CI)
Age (years), mean (SD)	74.94 (2.65)	75.84 (3.92)	.10	–0.260 (–0.564 to 0.045)
Sex n (%)			.19^d^	
Male	36 (50)	40 (40)		
Female	36 (50)	60 (60)		
BMI (kg/m^2^), mean (SD)	25.55 (2.87)	27.90 (4.10)	<.001	–0.647 (–0.956 to –0.335)
HGS^e^ (kg), mean (SD)	31.92 (9.70)	27.18 (9.41)	.002	0.497 (0.188 to 0.803)
SCPT^f^ (W), mean (SD)	235.82 (47.59)	212.23 (44.72)	.001	0.513 (0.205 to 0.820)
TUG^g^ (s), mean (SD)	7.40 (0.98)	8.80 (1.59)	<.001	–1.024 (–1.344 to –0.700)
SPPB^h^ total score, mean (SD)	11.51 (0.71)	10.82 (1.16)	<.001	0.696 (0.384 to 1.007)
5CRT stopwatch duration (s), mean (SD)	10.38 (1.83)	12.49 (2.85)	<.001	–0.852 (–1.167 to –0.535)
4mGS^i^ (s), mean (SD)	2.41 (0.31)	2.81 (0.46)	<.001	–1.008 (–1.328 to –0.686)
‍4mGS (m/s), mean (SD)	1.70 (0.23)	1.46 (0.22)	<.001	1.046 (0.722 to 1.367)
6minWT^j^ distance (m), mean (SD)	499.73 (61.27)	430.25 (59.02)	<.001	1.159 (0.830 to 1.484)
‍Semitandem stand (points), mean (SD)	1 (0)	1 (0)	*>*.99^k^	
‍Tandem stand (points), mean (SD)	1.85 (0.36)	1.83 (0.43)	>.99^l^	
CMJ^m^ height (cm), mean (SD)	12.86 (4.47)^n^	9.05 (3.84)^o^	<.001	0.926 (0.572 to 1.283)
CMJ power (W), mean (SD)	1952.28 (538.34)^n^	1766.56 (446.85)^o^	.03	0.383 (0.041 to 0.724)

a DTW: dynamic time warping.

b 5CRT: Five Times Chair Rise Test.

c *P* values are derived from independent samples *t* tests (highly significant: *P*<.001, significant: *P*<.01, marginally significant: *P*<.05),

d Derived from Pearson chi-squared test for categorical variables: *χ*2_1_=1.697.

e HGS: Handgrip Strength.

f SCPT: Stair Climb Power Test.

g TUG: Timed “Up & Go.”

h SPPB: Short Physical Performance Battery.

i 4mGS: 4-meter Gait Speed test.

j 6minWT: 6-minute Walking Test.

k Derived from Mann-Whitney *U* test for nonparametric data: *U*=3600.

l Derived from Mann-Whitney *U* test for nonparametric data: *U*=3599.

m CMJ: Countermovement Jump.

n n=55 for cluster 1.

o n=86 for cluster 2.

**Table 7.** Demographic and physical performance outcomes for participants in DTW^a^-defined 5CRT^b^ movement clusters (clusters 1 and 2) according to Meyer et al [10].

Variables at t2 (24 months)	Cluster 1 (n=72)	Cluster 2 (n=100)	*P^c^* value	Effect size, Cohen *d* (95% CI)
Age (years)	76.42 (2.70)	77.30 (3.85)	.09	–0.264 (–0.568 to 0.040)
Sex, n (%)			.19^d^	
Male	36 (50)	40 (40)		
Female	36 (50)	60 (60)		
BMI (kg/m^2^), mean (SD)	25.60 (2.80)	27.850 (3.95)	<.001	–0.641 (–0.951to –0.330)
HGS^e^ (kg), mean (SD)	27.64 (9.84)	23.21 (9.12)	.003	0.471 (0.163-0.777)
SCPT^f^ (W), mean (SD)	222.67 (48.01)	193.05 (42.56)	<.001	0.659 (0.348-0.969)
TUG^g^ (s), mean (SD)	7.55 (0.97)	9.52 (1.53)	<.001	–1.487 (–1.827 to –1.144)
SPPB^h^ total score, mean (SD)	11.69 (0.57)	10.59 (1.08)	<.001	1.219 (0.888-1.547)
5CRT stopwatch duration (s), mean (SD)	9.84 (1.42)	12.60 (2.48)	<.001	-1.311 (-1.643,‐0.976)
4mGS^i^ (s), mean (SD)	1.68 (0.19)	1.38 (0.20)	<.001	1.495 (1.152-1.836)
‍4mGS (m/s), mean (SD)	2.42 (0.28)	2.95 (0.44)	<.001	–1.411 (–1.747to –1.071)
6minWT^j^ distance (m), mean (SD)	481.35 (65.99)^k^	411.74 (60.08)^l^	<.001	1.111 (0.777-1.443)
‍Semitandem stand (points), mean (SD)	1 (0)^m^	0.96 (0.20)^n^	.09^o^	
‍Tandem stand (points), mean (SD)	1.86 (0.39)^m^	1.62 (0.65)^n^	.008^p^	
CMJ^q^ height (cm)	—^r^	—	—	—
CMJ power (W)	—	—	—	—

a DTW: dynamic time warping.

b 5CRT: Five Times Chair Rise Test.

c *P* values are derived from independent samples *t* tests (highly significant: *P*<.001, significant: *P*<.01, marginally significant: *P*<.05).

d Derived from Pearson chi-squared test for categorical variables: *χ*2_1_=1.697.

e HGS: Handgrip Strength.

f SCPT: Stair Climb Power Test.

g TUG: Timed “Up & Go.”

h SPPB: Short Physical Performance Battery.

i 4mGS: 4-meter Gait Speed test.

j 6minWT: 6-minute Walking Test.

k n=69 for cluster 1.

l n=95 for cluster 2.

m n=72 for cluster 1.

n n=100 for cluster 2.

o Derived from Mann-Whitney *U* test for nonparametric data: *U*=3456.

p Derived from Mann-Whitney *U* test for nonparametric data: *U*=2980.

q CMJ: Countermovement Jump.

r Not available.

The t2 clusters differed significantly in several parameters (Table 7). To understand the effect of 5CRT duration, the clusters were divided by each score category, as presented by Guralnik et al [8]. Figure 4 shows the barycenter for each resulting dataset. Note that cluster 1 does not contain any participants with a 5CRT duration greater than 13 seconds, while cluster 2 contains participants in all score categories.

To sum up, 2 functional movement clusters (cluster 1 and 2) were identified among older adults performing the 5CRT (Figures 3 and 4): cluster 1 was characterized by faster chair rise times and lower vertical GRFs during stabilization, reflecting better physical performance and postural control. Conversely, cluster 2 exhibited slower performance times and elevated forces during stabilization, suggesting compromised mobility and potential balance limitations. These clusters revealed underlying movement pattern differences that traditional stopwatch timing could not detect, demonstrating the enhanced sensitivity of ML-based time-series analysis.

**Figure 4.**
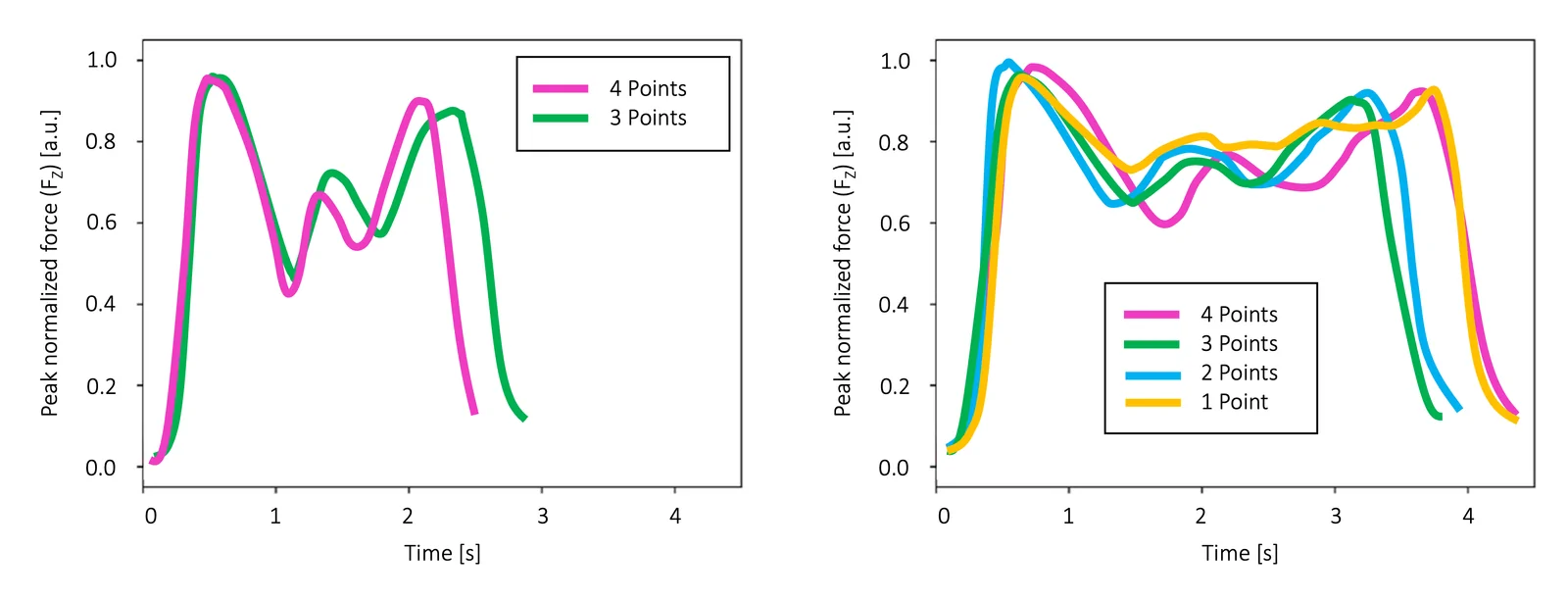
Barycenter of subdatasets created by splitting cluster 1 (left) and cluster 2 (right) datasets according to Meyer et al [10] based on each scored category according to Guralnik et al [8].

### Associations of ML-Based 5CRT Clusters With Clinical Physical Performance Tests

The demographic and physical performance characteristics of the 2 5CRT movement clusters identified by the ML-driven clustering approach proposed by Meyer et al [10] are presented in Tables 5-7 for each of the 3 assessment time points (t0, t1, and t2).

At baseline (t0; Table 5), age and sex distribution did not differ significantly between cluster 1 and cluster 2: participants in cluster 1 and cluster 2 were of comparable age (mean 74.40, SD 2.70 years vs mean 75.30, SD 3.90 years; Cohen *d*=–0.259; *P*=.09) and showed no significant difference in sex distribution (n=36, 50% male vs n=40, 40% male; *P*=.19). In contrast, both clusters differ in anthropometry and physical performance. Cluster 1 participants had a significantly lower BMI compared to cluster 2 participants (mean 25.64, SD 2.90 kg/m^2^ vs mean 27.97, SD 4.09 kg/m^2^; Cohen *d*=–0.640; *P*<.001). Measures of muscle strength and power were higher in cluster 1: participants in cluster 1 exhibited significantly higher HGS (mean 32.41, SD 9.88 kg vs mean 28.07, SD 9.60 kg; Cohen *d*=0.447; *P*=.004) and SCPT values (mean 236.15, SD 51.30 W vs mean 214.66, SD 47 W; Cohen *d*=0.440; *P*=.005) compared to cluster 2. Both clusters also differ in mobility and global physical function: compared to cluster 2, cluster 1 participants had faster TUG times (mean 7.42, SD 0.95 seconds vs mean 8.74, SD 1.44 seconds; Cohen *d*=–1.047; *P*<.001) and higher SPPB total scores (mean 11.35, SD 0.75 versus mean 10.93, SD 1.10, Cohen *d*=0.429; *P*=.006). The 5CRT performance showed differences between cluster 1 and cluster 2: the 5CRT stopwatch duration was significantly shorter in cluster 1 participants compared to cluster 2 participants (mean 11.15, SD 2.07 seconds vs mean 12.80, SD 3.30 seconds; Cohen *d*=–0.581; *P*<.001). Habitual gait speed, assessed via 4mGS, was faster in cluster 1 compared to cluster 2 (mean 2.44, SD 0.30 seconds vs mean 2.87, SD 0.42 seconds, Cohen *d*=–1.167; *P*<.001 and mean 1.66, SD 0.22 m/s vs mean 1.42, SD 0.20 m/s; Cohen *d*=1.190; *P*<.001). 6minWT distance was higher in cluster 1 (mean 490, SD 60.27 m vs mean 421.21, SD 60.34 m; Cohen *d*=1.141; *P*<.001). Balance measures showed near ceiling in both clusters: the semi-tandem stand revealed mean 1 (SD 0) points for cluster 1 participants vs mean 0.99 (SD 0.10) points for cluster 2 participants (*P*=.40). In contrast, tandem stance showed mean 1.99, (SD 0.12) points in cluster 1 and mean 1.92 (SD 0.32) points in cluster 2 (*P*=.05). Explosive lower-limb performance, assessed by CMJ, also differed between cluster 1 and cluster 2 at baseline: cluster 1 participants showed higher CMJ height (mean 13.07, SD 4.71 cm vs mean 9.55, SD 4.23 cm; Cohen *d*=0.793; *P*<.001) and power (mean 2050.17, SD 577.12 W versus mean 1816.86, SD 482.32 W, Cohen *d*=0.445; *P*<.007) than cluster 2 participants.

After 6 months (t1; Table 6), the patterns observed at baseline (t0) continue to show similar demographic profiles but differences in physical performance. Age and sex distribution did not differ significantly between cluster 1 and cluster 2: participants in cluster 1 and cluster 2 were of comparable age (mean 74.94, SD 2.65 years vs mean 75.84, SD 3.92 years, Cohen *d*=–.260; *P*=.09) and showed no significant difference in sex distribution (50% [36] male vs 40% [40] male; *P*=.19). Cluster 1 participants had a significantly lower BMI compared to cluster 2 participants (mean 25.55, SD 2.87 kg/m^2^ vs mean 27.90, SD 4.10 kg/m^2^, Cohen *d*=–0.647; *P*<.001). At t1, measures of muscle strength and power were still higher in cluster 1: participants in cluster 1 showed significantly higher HGS (mean 31.92, SD 9.70 kg vs mean 27.18, SD 9.41 kg, Cohen *d*=0.497; *P*=.002) and SCPT values (mean 235.82, SD 47.59 W vs mean 212.23, SD 44.72 W, Cohen *d*=0.513; *P*=.001) compared to cluster 2. Both clusters also differ in mobility and global physical function: compared to cluster 2, cluster 1 participants still had faster TUG times (mean 7.40, SD 0.98 s vs mean 8.80, SD 1.59 s, Cohen *d*=–1.024; *P*<.001) and higher SPPB total scores (mean 11.51, SD 0.71 vs mean 10.82, SD 1.16, Cohen *d*=–0.696; *P*<.001). The 5CRT performance still showed differences between cluster 1 and cluster 2 at t1: the 5CRT stopwatch duration was significantly short in cluster 1 participants compared to cluster 2 participants (mean 10.38, SD 1.83 seconds vs mean 12.49, SD 2.85 seconds, Cohen *d*=–0.852; *P*<.001). 4mGS was faster in cluster 1 compared to cluster 2 (mean 2.41, SD 0.31 seconds vs mean 2.81, SD 0.46 s, Cohen *d*=–1.008; *P*<.001 and mean 1.70, SD 0.23 m/s vs mean 1.46, SD 0.22 m/s, Cohen *d*=1.046; *P*<.001). In addition, 6-minute walk test distance was higher in cluster 1 (mean 499.73, SD 61.27 m vs mean 430.25, SD 59.02 m, Cohen *d*=1.159; *P*<.001). Balance measures also showed near ceiling at t1 in both clusters: The semi-tandem stand revealed mean 1 (SD 0) points for cluster 1 and cluster 2 participants (*P>*.99). In contrast, tandem stance showed mean 1.85 (SD 0.36) points in cluster 1 and mean 1.83 (SD 0.43) points in cluster 2 (*P*>.99). Explosive lower-limb performance also differed cluster 1 and cluster 2 at t1: compared to cluster 2, cluster 1 participants showed higher CMJ height (mean 12.86, SD 4.47 cm vs mean 9.05, SD 3.84 cm, Cohen *d*=0.926; *P*<.001) and power (mean 1952.28, SD 538.34 W vs mean 1766.56, SD 446.85 W, Cohen *d*=0.383; *P*=.03).

After 24 months (t2; Table 7), age and sex distributions remained similar across cluster 1 and cluster 2 (mean age 76.42, SD 2.70 years vs mean 77.30, SD 3.85 years, Cohen *d*=–0.264; *P*=.09; sex: n=36, 50% male vs n=40, 40% male; *P*=.19). The differences in BMI persisted as moderately higher in cluster 2 participants (mean 25.60, SD 2.80 kg/m² vs mean 27.85, SD 3.95 kg/m², Cohen *d*=–0.641; *P*<.001). At t2, several functional differences between cluster 1 and cluster 2 existed: cluster 1 participants maintained significantly higher HGS (mean 27.64, SD 9.84 kg vs mean 23.21, SD 9.12 kg, Cohen *d*=0.471; *P*=.003) and SCPT values (mean 222.67, SD 48.01 W vs mean 193.05, SD 42.56 W, Cohen *d*=0.659; *P*<.001). In addition, mobility and global performance differences further increased: TUG times remained faster in cluster 1 (mean 7.55, SD 0.97 seconds vs mean 9.52, SD 1.53 seconds, Cohen *d*=–1.487; *P*<.001), and SPPB total scores were higher (mean 11.69, SD 0.57 vs mean 10.59, SD 1.08, Cohen *d*=1.219; *P*<.001). The 5CRT performance showed differences between cluster 1 and cluster 2: the 5CRT stopwatch duration was significantly short in cluster 1 participants compared to cluster 2 participants (mean 9.84, SD 1.42 seconds vs mean 12.60, SD 2.48 seconds, Cohen *d*=–1.311; *P*<.001). Habitual gait speed, assessed via 4mGS, was faster in cluster 1 compared to cluster 2 (mean 1.68, SD 0.19 seconds vs mean 1.38, SD 0.20 seconds, Cohen *d*=1.495; *P*<.001 and mean 2.42, SD 0.28 m/s vs mean 2.95, SD 0.44 m/s, Cohen *d*=–1.411; *P*<.001). 6minWT distance remained higher in cluster 1 (mean 481.35, SD 65.99 m vs mean 411.74, SD 60.08 m, Cohen *d*=1.111; *P*<.001). Balance measures showed a more nuanced pattern. Semi-tandem stance remained near ceiling in both clusters, with only a nonsignificant trend toward a higher score in cluster 1 (mean 1, SD 0 vs mean 0.96, SD 0.20; *P*=.09). In contrast, tandem stance revealed a significant advantage for cluster 1 participants (mean 1.86, SD 0.39 vs mean 1.62, SD 0.65; *P*=.008).

CMJ height and CMJ power were not assessed at t2.

Figure 5 shows a radar plot of Cohen *d* effect sizes of each assessment and each variable at baseline (t0), 6-month (t1), and 24-month (t2) follow-up. The effect sizes are plotted relative to a central zero line, representing no difference between cluster 1 and cluster 2. Concentric rings correspond to conventional interpretation thresholds for negligible (0.00-0.19), small (0.20-0.49), moderate (0.50-0.79), and large (≥ 0.8) effects.

**Figure 5.**
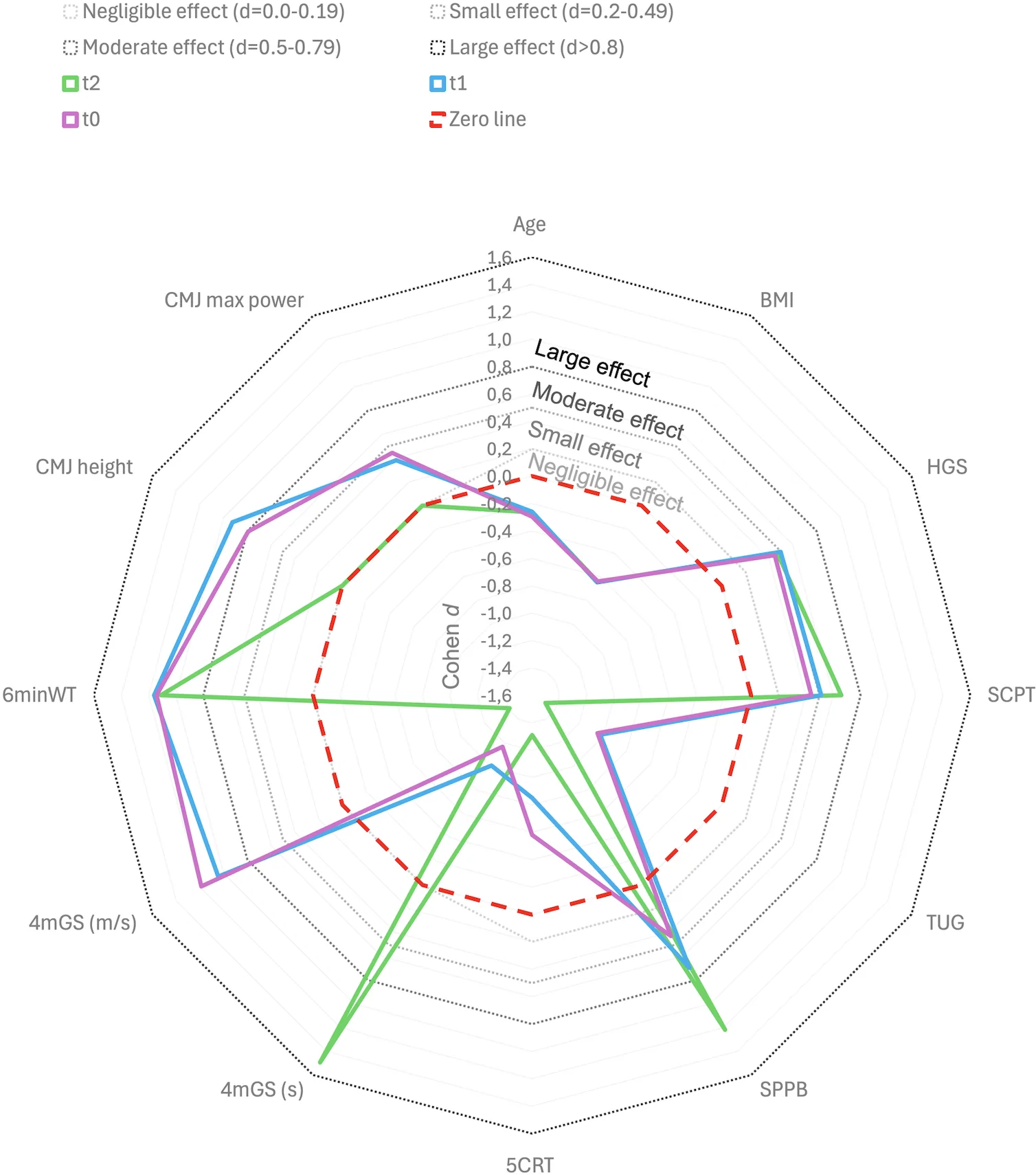
The radar plot shows Cohen *d* effect sizes for various assessments (age, BMI, Handgrip Strength test, Stair Climb Power Test, Timed “Up and Go” test, Short Physical Performance Battery, Five Times Chair Rise Test, 4-meter Gait Speed, 6-minute Walking Test, Countermovement Jump height and Countermovement Jump power) at three time points (t0=baseline, t1=6 months, and t2=24 months) in relation to the zero line (red). Concentric rings represent thresholds for negligible, small, moderate, and large effects. 4mGS: 4-meter Gait Speed; 5CRT: Five Times Chair Rise Test; 6minWT: 6-minute Walking Test; CMJ: Countermovement Jump; HGST: Handgrip Strength test; SCPT: Stair Climb Power Test; SPPB: Short Physical Performance Battery; TUG: Timed “Up & Go”.

Across all 3 time points, age showed small negative effects, indicating that participants in cluster 1 were slightly younger. BMI consistently showed moderate negative effects, reflecting a higher BMI in cluster 2 at all time points. In contrast, functional and performance measures clearly fall outside the negligible range and generally favor cluster 1. At t0, the effect sizes were small for HGS (Cohen *d*=0.447), SCPT (Cohen *d*=0.440), SPPB (Cohen *d*=0.429), and CMJ height and power (Cohen *d*=0.445, Cohen *d*=0.793). Mobility and gait-related outcomes showed large between-cluster differences at baseline: TUG (Cohen *d*=-1.047), 4mGS in seconds (Cohen *d*=–1.167), 4mGS in m/s (Cohen *d*=1.190), and 6minWT (Cohen *d*=1.141). There was also a moderate-to-large effect for 5CRT duration (Cohen *d*=–0.581). These effects indicate that cluster 1 had higher strength, mobility, gait speed, endurance, and jump performance than cluster 2 at baseline.

After 6 months (t1), this pattern persisted and more pronounced for several measures. Effect sizes were small to moderate for HGS (Cohen *d*=0.497), SCPT (Cohen *d*=0.513), and CMJ power (Cohen *d*=0.383). They increased to moderate for the SPPB (Cohen *d*=0.696). CMJ height showed a large effect size (Cohen *d*=0.926), which further indicates higher explosive lower-limb power in cluster 1. Mobility and gait outcomes again exhibited large effects: TUG (Cohen *d*=–1.024), 4mGS in seconds (Cohen *d*=–1.008), 4mGS in m/s (Cohen *d*=1.046), 6minWT (Cohen *d*=1.159), and 5CRT duration (Cohen *d*=–0.852). Overall, the radar plot at t1 shows a widening separation between both clusters for key functional measures, particularly for mobility, gait speed, endurance, and CMJ height.

After 24 months (t2), the divergence in performance between cluster 1 and cluster 2 is even more pronounced for several outcomes. HGS (Cohen *d*=0.471) remained in the small-to-moderate range, while SCPT showed a moderate effect (Cohen *d*=0.659). SPPB (Cohen *d*=1.219), TUG (Cohen *d*=–1.487), 5CRT duration (Cohen *d*=–1.311), 4mGS in seconds (Cohen *d*=1.495), 4mGS in m/s (Cohen *d*=–1.411), and 6minWT (Cohen *d*=1.111) demonstrated large effect sizes. This indicates substantial, clinically meaningful differences in lower extremity performance, balance, gait speed, and endurance favoring cluster 1. CMJ height and CMJ power were not assessed at t2, as shown by the missing values on the radar plot at t2.

In summary, cluster 1 demonstrated higher functional performance at all 3 time points (t0, t1, and t2). Effect sizes indicated progressively larger differences compared to cluster 2. The largest differences were observed in mobility- and gait-related outcomes such as TUG, 4mGS, 6minWT, SPPB, and 5CRT. These outcomes showed the highest effect sizes and the greatest increases from baseline (t0) to the 24-month follow-up assessment (t2).

### Added Value of ML-Based Classification Compared to Conventional Stopwatch Timing

To evaluate whether ML-based 5CRT movement pattern classification provides clinically meaningful differentiation of functional status beyond conventional stopwatch-based 5CRT timing, Figure 6 shows the distribution of participants classified by conventional, stopwatch-derived 5CRT scoring (left panel) and ML-based DTW cluster assignment (right panel). Using stopwatch-based thresholds according to Guralnik et al [8] (Table 2), most participants fell into the “good” performance category (3‐4 points), while only a minority were in the impaired range (≤2 points). This suggests homogeneity and relatively preserved function in the cohort when interpreted by conventional timing alone.

However, the ML-based classification revealed substantial heterogeneity within the same stopwatch categories. While all cluster 1 participants were in the higher score ranges, cluster 2 participants spanned all performance levels, including many who scored 3‐4 points by stopwatch criteria (Figure 6, right). Notably, these cluster 2 individuals exhibited elevated stabilization forces, slower mobility (TUG), and reduced endurance (6minWT), indicating clinically relevant impairments not reflected in their stopwatch score.

**Figure 6.**
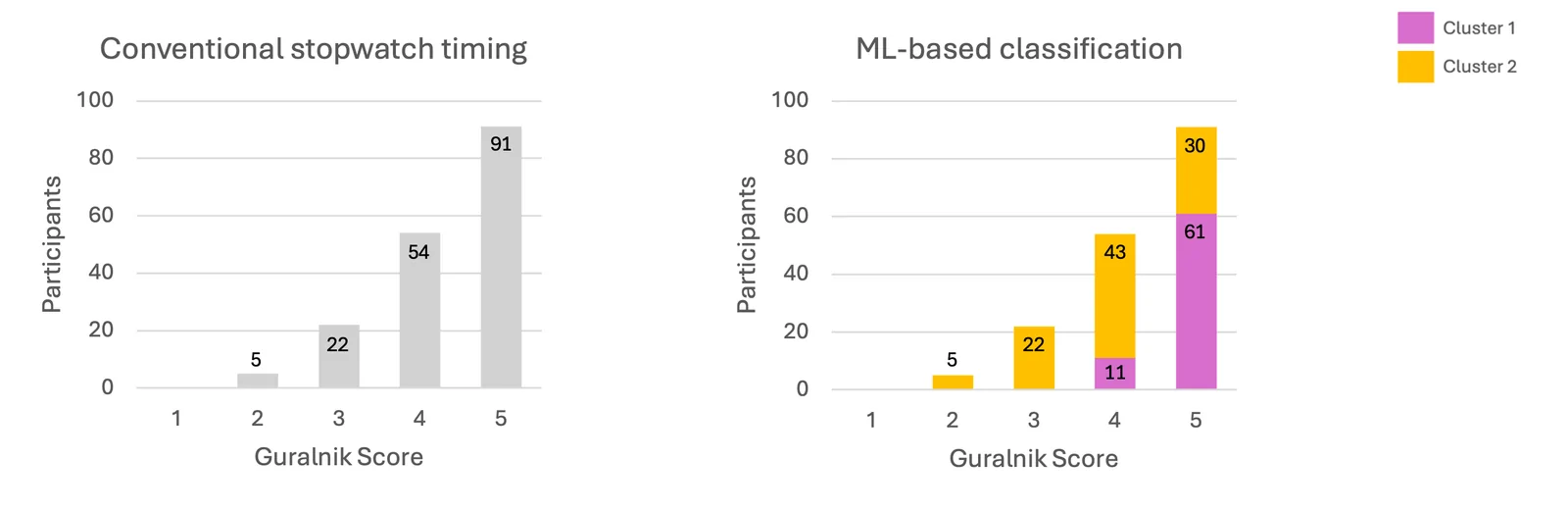
Comparison of conventional stopwatch-based Five Times Chair Rise Test scoring (left) and machine learning–based movement classification (right). While the conventional stopwatch-based Five Times Chair Rise Test scoring condensed most participants into high-performance categories (3-4 points), the machine learning–based clustering revealed hidden heterogeneity, separating participants into 2 clusters with clearly distinct functional profiles.

## Discussion

### Principal Findings

This study highlights the value of advanced, ML-based biomechanical analysis, specifically, DTW combined with k-means clustering, in identifying functional performance profiles among community-dwelling older adults performing the 5CRT. The results are discussed in the order of the predefined study aims.

### Classification of 5CRT Movement Patterns Using ML-Based Clustering

The first study aim was to classify 5CRT movement patterns using DTW and k-means clustering, using the workflow established by Meyer et al [10] to ensure methodological comparability. The findings confirm that ML-based pattern recognition can differentiate between clusters of chair rise execution. Consistent with Meyer et al [10], 2 distinct movement clusters were identified at the 24-month follow-up assessment (t2). All participants lived in the community, were free from major acute morbidities, had to be able to climb 10 flights of stairs without difficulty, and complete a TUG in less than 20 seconds. Despite this high baseline functional level, the ML-based pattern recognition detected differences in the shape and temporal dynamics based on 5CRT execution. Cluster 1 was characterized by faster mean 5CRT execution time of 9.84 (SD 1.42) seconds (Table 6) and lower stabilization forces (Figures 3 and 4). Cluster 2 exhibited 5CRT execution with a mean duration of 12.60 (SD 2.48) seconds (Table 6) and increased vertical GRFs during stabilization (Figures 3 and 4).

Although clustering was performed at t2, the functional differences between cluster 1 and cluster 2 participants were already evident at baseline and generally widened over the 24-month period (Tables 5-7 and Figure 5). This pattern suggests that the 5CRT movement patterns captured by the DTW-based approach may reflect individual differences in movement execution rather than purely transient variability. However, because cluster assignment was based on t2 data, these findings should be interpreted as descriptive stratification rather than evidence of distinct baseline trajectories or causal mechanisms. The identified clusters may represent a functional performance profile that is stable over time, but this remains hypothesis-generating and requires prospective validation.

Importantly, this multidimensional approach revealed functional heterogeneity within a population of community-dwelling older adults who were otherwise classified as healthy based on conventional criteria. Stopwatch timing produced a concentrated score range, with most participants falling into a high-functioning category (Figure 6), whereas DTW-based clustering identified subgroups with differing levels of mobility, strength, and postural control.

To sum up, the DTW-based clustering approach is a useful tool for identifying movement patterns in the 5CRT. However, the results may be influenced by preprocessing such as segmentation, resampling, and normalization. These steps can influence the input structure, thereby impacting the subsequent cluster solution. Additionally, different cluster sizes may lead to frequent movement patterns dominating the analysis, thereby underrepresenting less frequent patterns. Recent clinical movement studies [21] have demonstrated that data distribution and balancing decisions can substantially influence model-derived classifications, especially in small or heterogeneous datasets. Consequently, these findings should be interpreted as exploratory, and future work should assess the robustness of the results across a range of alternative preprocessing and sampling strategies.

### Association Between Movement Pattern Clusters and Functional Performance Indicators

The second study aim focused on quantifying the associations between the ML-based movement clusters and established clinical outcomes. Across the baseline (t0), 6-month (t1), and 24-month (t2) follow-up assessments, participants in cluster 1 consistently outperformed those in cluster 2 on key performance indicators such as lower extremity strength (HGS), leg power (SCPT), mobility and gait (TUG and 4mGS), endurance (6minWT), global functional status (SPPB), and explosive capacity (CMJ power and height; Tables 5-7). These differences between both clusters were already present at baseline and generally increased over time, indicating diverging functional trajectories between cluster 1 and cluster 2.

The separation between cluster 1 and cluster 2 participants was most evident at t2, where effect sizes for 4mGS, TUG, and 6minWT reached the moderate-to-large range, pointing to disparities in mobility and endurance despite the cohort’s initially high functional status. The pronounced separation at t2 is expected, as the clusters were assigned exclusively on t2 data using DTW-based k-means clustering, resulting in an inherent maximization of intercluster differences at this time point and strengthening the correlation between ML-based movement patterns and clinically meaningful functional performances.

In parallel, BMI and body weight were consistently higher in cluster 2 at all time points, with differences becoming particularly evident by t2. This pattern suggests that the ML-based movement clusters do not merely reflect transient performance fluctuations but align with broader anthropometric risk profiles that are known to coevolve with mobility decline and incident disability in older adults [22].

From a mechanistic point of view, these characteristics are plausible, but several interpretations remain possible. The 5CRT combines lower-limb power, postural control, and interlimb coordination [8,9]. The temporal dynamics of movement, as captured by DTW-based clustering, may encode compensatory strategies [10]. These strategies could include prolonged transition phases, altered sequencing, or pace variability that are not fully reflected by total task duration.

At the same time, the consistent superiority of cluster 1 across all functional domains suggests that the clusters may primarily reflect overall functional capacity or a latent fitness axis, rather than fundamentally distinct biomechanical strategies. In this view, DTW-based clustering may provide a more granular stratification of performance levels rather than identifying entirely novel movement phenotypes. This interpretation is consistent with the observation that cluster assignment is associated with stopwatch-based 5CRT duration.

The difference in BMI and body weight between cluster 1 and cluster 2 further supports a link between movement pattern quality, body composition, and functional aging. A higher BMI has been associated with adverse changes in body composition, reduced muscle quality, and increased risk of mobility disability and mortality [22].

In summary, the longitudinal association between ML-defined movement clusters and a broad spectrum of functional indicators suggests that temporal characteristics of chair-rise performance may be related to clinically relevant differences in functional aging. These results suggest that movement quality, as quantified by ML, may provide useful information about neuromuscular reserve and emerging frailty in otherwise high-functioning older adults.

### Added Value of ML-Based Classification Compared to Conventional Stopwatch Timing

The third study aim was to evaluate the added clinical value offered by ML-based movement pattern classification beyond conventional stopwatch-based timings. Distributional analysis revealed that stopwatch timing produced a more concentrated score range, with most participants falling within the high-functioning category (Figure 6). In contrast, DTW-based clustering revealed significant heterogeneity (Figure 6): cluster 2 participants displayed subclinical impairments in mobility, strength, and postural control despite achieving “good” stopwatch scores (Tables 5-7 and Figure 6). These discrepancies may highlight the value of sensor-based analytical methods in identifying latent deficits, particularly those related to compensatory strategies and stabilization, thus enabling more granular risk assessment and targeted early intervention.

From a clinical perspective, this approach may facilitate a transition toward personalized movement phenotyping for risk stratification, prognosis, and rehabilitation planning in older adults. Integrating ML-based biomechanical metrics with comprehensive geriatric assessments provides a more nuanced understanding of the aging process and functional decline trajectories that are not captured by traditional methods solely.

Our findings may support further development of precision geriatric assessment, in which multidimensional data and ML could contribute to the early identification of individuals at risk. This approach may help refine intervention strategies and support monitoring of mobility and independence in aging populations.

However, the incremental predictive value of cluster assignment beyond conventional 5CRT duration was not formally tested in this study. While the patterns suggest that sensor-based analytics may provide additional granularity, particularly for detecting subclinical deficits related to compensatory strategies and stabilization, this remains suggestive rather than proven. Formal analyses of incremental predictive value (eg, regression models with and without cluster assignment) are needed to establish whether ML-based clustering offers clinically meaningful added value beyond simple timing.

### Limitations

Despite the strengths of a large cohort and robust biomechanical assessments, several limitations warrant consideration. The study sample consisted of relatively healthy community-dwelling older adults recruited from a specific geographical region in Germany, which may restrict the generalizability of our findings to more diverse or frailer populations. In addition, attrition over the 24-month period (including withdrawals and missing data) reduced the analytical sample size, potentially introducing bias toward healthier individuals who remained in the study.

Clusters were defined exclusively on the final time point (t2) data using k-means with DTW, which is sensitive to initial conditions, preprocessing choices, and cluster size imbalance. The pronounced separation observed at t2 is expected and should not be interpreted as independent evidence of longitudinal divergence. Instead, these discrepancies likely mirror the clustering procedure itself, which aimed to optimize inter-group separation at t2. Consequently, the observed variations at t0 and t1 may be indicative of post hoc stratification rather than genuinely distinct baseline trajectories. Future studies should use baseline clustering or temporal clustering approaches to evaluate prospective subgroup development. In addition, bootstrapping, stability analyses, or cluster consistency checks across time points as well as testing reproducibility in an independent cohort should be addressed in future work.

Furthermore, the strong alignment of cluster assignment with established functional variables raises the possibility that the clusters primarily reflect overall functional capacity rather than distinct movement strategies. In this study, it was not formally tested whether cluster assignment provides incremental predictive value beyond conventional metrics. These findings should therefore be interpreted as exploratory.

A limitation of this study is that the clustering approach did not explicitly model intraindividual changes across the 5 chair rises such as fatigue-, adaptation-, or motor control–related dynamics. In the data reported by Meyer et al [10], the first chair rise was generally consistent with the cluster assigned to the remaining repetitions, but subtle repetition-dependent effects may still not have been fully captured. Future studies should therefore consider repetition-aware or longitudinal approaches to investigate intraindividual changes in greater detail.

Although the use of a height-adjustable chair at t2 improved test standardization, slight deviations in chair height or leg position might still have influenced performance measures. Furthermore, the current setup using a force plate and depth camera offers high precision but may have limited scalability in everyday clinical environments. Wearable sensor technologies could serve as feasible alternatives for future large-scale or home-based assessments.

Finally, while ML-based clustering and DTW techniques enhanced sensitivity to subtle movement variations, broader validation across different sensor modalities, populations, and settings will be necessary to confirm their clinical utility and reproducibility. Future studies should also address psychosocial factors, environmental influences, and acceptability of such technology-driven assessments in routine geriatric care.

Claims regarding added clinical value should be considered preliminary.

### Conclusions

This study demonstrates that ML-based analysis of dynamic chair rise movements using DTW and k-means clustering successfully identifies subgroups of older adults that cannot be discerned from conventional stopwatch timing metrics alone. These clusters exhibit significant differences in physical function, frailty, and mobility, as confirmed by comprehensive clinical assessments. The findings demonstrate that temporal characteristics of chair-rise performance provide information beyond total test duration. This study highlights the potential of advanced biomechanical analytics to improve geriatric screening and support the planning of personalized interventions. Further validation in larger, more diverse cohorts, and the integration of sensor-based assessments into routine practice, could optimize functional assessment and promote healthy aging at a population level.

## Abbreviations

4mGS: 4-meter Gait Speed
5CRT: Five Times Chair Rise Test
6minWT: 6-minute Walking Test
BT: Balance Test
CMJ: Countermovement Jump
COPD: chronic obstructive pulmonary disease
DBA: DTW Barycenter Averaging
DTW: dynamic time warping
GRF: ground reaction force
HGS: Handgrip Strength
ML: machine learning
SCPT: Stair Climb Power Test
SPPB: Short Physical Performance Battery
TUG: Timed "Up & Go"
